# The influence of applying insurance medicine guidelines for depression on disability assessments

**DOI:** 10.1186/1756-0500-6-225

**Published:** 2013-06-07

**Authors:** Antonius JM Schellart, Feico Zwerver, Johannes R Anema, Allard J Van derBeek

**Affiliations:** 1 Department of Public and Occupational Health, EMGO Institute for Health and Care Research, VU University Medical Center, Amsterdam, The Netherlands; 2 Research Center for Insurance Medicine, AMC-UMCG-UWV-VUmc, Amsterdam, The Netherlands; 3 Dutch National Institute for Employee Benefits Schemes, Amsterdam, The Netherlands

## Abstract

**Background:**

In the current study we report on the effects of an implementation strategy in the form of a training programme on the assessed work limitations of a client with depression by insurance physicians (IPs) participating in a RCT. These assessed work limitations of a client were in the form of scores on the List of Functional Abilities (LFA).

**Method:**

We conducted a randomised controlled trial (RCT) for IPs in which we compared the intervention of a specially developed training programme with the usual methods of implementation and training currently used. The outcome was the mean sum score and the inter-rater reliability (Intraclass Correlation Coefficient, ICC) of the LFA scores. These LFA scores were scored by the IPs participating in the RCT for the work limitations of the cases presented in different videos, two videos before the training and two after the training of the intervention group.

**Results:**

At baseline, the intervention group (IG) consisted of 21 IPs and the control group (CG) of 19. For one participant of the IG and for one of the CG the LFAs of the two case reports after training were not available. Before training the sum scores for the first case report did not differ significantly between the groups, while the mean sum score was higher in the IG than in the CG for the second case report. For both case reports after training a higher score was found in the IG than in the CG. The inter-rater reliability measured for the two case reports before training was about the same in the IG and the CG: 0.64 and 0.65, respectively. For the two case reports after training, the ICC was higher in the IG than in the CG: 0.69 and 0.54, respectively. This difference was not significant however.

**Conclusion:**

It would appear that the implementation of a specially designed training programme on guidelines for depression may lead to greater inter-rater reliability in the assessments by insurance physicians of the work limitations of clients with depression. It is, however, important to note that insurance physicians who receive training may find more work limitations than those who do not.

**Trial registration:**

Netherlands’ Trial Register NTR1863

## Background

### Insurance medicine in the Netherlands

The Dutch National Institute for Employee Benefit Schemes (the Institute) administers the eligibility of sick employees for a benefit under the Work and Income (Capacity for Work) Act (WIA). 900 Insurance physicians (IP) are employed at the Institute, approx. 450 of who perform disability assessments under the WIA [1]. On average, these insurance physicians are 50 years old, 59% is men, they have approx. 16 years experience as insurance physician, approx. 86% is specialized in insurance medicine, 15% also has another extra medical speciality, and approx. 60% works full-time. They perform an average of 9 disability assessments per week, assessing employees with all types of diseases [1]. Employees who are on sick leave for two years can claim a disability benefit through the Institute. Such an employee becomes a client of the Institute. The clients’ claim is assessed by an IP at a front office of the Institute. In this assessment, that is called the work disability assessment, the client’s work limitations and abilities are defined. The IP writes his or her findings down in a medical work disability report and fills in a List of Functional Abilities (LFA) [2]. On average, an IP uses approximately two hours for a complete work disability assessment. One hour for the assessment interview, and one hour for writing the report. Subsequently, a labour expert matches the client’s work abilities as have been defined in the LFA, with the functional demands of (theoretically) available jobs, resulting in a selection of jobs that the client should be able to perform, despite his/her work limitations. The client’s benefit, finally, is determined by the loss of income, caused by the difference in wages between that of the client’s initial job and the wages of the selected jobs.

### Guideline adherence and work limitations

We have previously investigated whether an implementation strategy that meets the needs of insurance physicians (IPs) leads to better adherence to guidelines than the usual implementation employed by the Dutch National Institute for Employee Benefits Schemes [3]. To this end we have developed a training programme using interventions that teach IPs how to apply the insurance medicine guidelines for depression [4] when performing assessments for work limitations. The efficacy of this implementation strategy was investigated in a randomised controlled trial (RCT), in which a group of IPs trained in applying the guidelines for depression were compared with a control group. We have demonstrated that IPs trained in applying the guidelines for depression scored significantly higher on guideline adherence and on knowledge of the guidelines for depression than IPs in the control group [5].

In the current study we report on the effects of this implementation strategy in the form of a training programme on the work limitations of a client with depression by insurance physicians participating in the RCT. As has been described above, these work limitations of a client, find by an IP, mainly determine the final rate of a clients´ disability and benefits. In general, the phenomenon of arbitrariness in the assessment of work limitations by IPs is socially undesirable. Therefore, it was needed to study the effect of the implementation strategy on the IPs´ way of assessing clients´ work limitations. Work limitations are recorded in scores on the LFA [2], and these scores represent a combination of the number as well as the severity of work limitations. The LFA is partly based on the International Classification of Functioning (ICF) [6]. The ICF has internationally been used for qualifying the level of functioning in disability assessments [7,8]. The following questions were therefore central to our research:

I. What is the influence of the training programme on the work limitations?

II. What is the influence of the training programme on the inter-rater reliability between the LFA scores of the participating IPs?

Previous research by Spanjer et al. [9] has shown that the more information about the client the IP has, the higher the number of work limitations the IP will find. A study by Schellart et al. [10] of inter-doctor variation between assessments by IPs found that greater adherence to the rules by IPs leads to a greater number of clients being assessed as the highest category of work disability. Based on these studies, our thoughts in the current study are that our intervention – a specific training programme on applying the guidelines for depression – will possibly lead to a more systematic overview of disorders and therefore to the finding of a higher number of work limitations in the RCT group than in the control group. We also think that our training programme may cause IPs to assess work limitations in a more uniform manner based on the information available. If this is indeed the case then the inter-rater reliability of the completed LFAs based on the same case reports should be greater in the intervention group than in the control group. Based on these thoughts we formulated the following hypotheses:

1) Training in guidelines for depression will result in to more work limitations, because adherence to the guidelines leads to a more complete overview of disorders and the resulting work limitations, based on the information available.

2) Training in guidelines for depression will result in higher inter-rater reliability between IPs: after following the training programme the IPs will assess work limitations in a more uniform manner.

## Methods

### Design

To determine the efficacy of a specially developed strategy for implementation of the guidelines for depression [4], we conducted a randomised controlled trial (RCT) in which we compared an intervention group with a control group. In this RCT we compared the intervention of a specially developed training programme with the usual methods of implementation and training currently in use by the social security agency.

The intervention was a training programme designed for IPs, in which they learnt to apply the guidelines for depression [3]. This programme, together with baseline and follow-up measurements, was integrated into a four-day postgraduate course located at the Netherlands School of Public and Occupational Health (NSPOH).

While the intervention group was trained in applying the guidelines for depression, the control group received an alternative programme of training in motivational interviewing that did not conflict with the intervention programme. The RCT took three days within a period of two weeks in March 2009. After the RCT ended, the control group received the same training as the intervention group, while the intervention group received the alternative programme. This was planned as the fourth day of the course, which was held three months later at the end of June 2009.

By using actors simulating four different case reports on video, we managed to create a laboratory setting in which we could measure the work disability assessments of clients with depression by each IP. In these videos the role of the client was played by four different actors, while the role of the IP was played by two ‘real’ IPs, independently selected for this purpose. The training programme was designed to be also applied in practice. The Ethics Committee of the VU University Medical Centre granted approval for the study design and the RCT was accepted by the Netherlands Trial Register under number NTR1863.

### Participants

In January 2009, IPs employed by the Institute were invited to take part in a postgraduate course in applying the guidelines for depression, given in the period from March to July 2009. The inclusion criteria were that individuals should be registered as insurance physicians, or still in training as such, and should be conducting disability assessments of clients as commissioned by the Institute. The NSPOH was responsible for enrolment of participants, who also provided written informed consent to take part in the study. 43 insurance physicians participated in the study.

The participants were allocated in order of registration to either the intervention group or the control group by using a random-sequence table. Participants who were not available on the planned dates were excluded from the trial. The participants were informed about the fact that the course was part of a research project, but they were not informed about the design of the entire project, i.e. the various measurements and the type of group they participated in.

### Data collection

Data were collected at the NSPOH during the period of the training course. At baseline (pre-intervention) and at follow-up (post-intervention) each IP assessed the work limitations of two clients, played by actors, who were presented separately on video. The actors played clients with depression, reconstructed from real case reports. The actors played their roles on the basis of extensive scripts, with room for improvisation. The videos showed the disability assessment encounter between a client (actor) and an independent IP (not a participant in the RCT), who had been briefed to perform the assessment in complete accordance with the guidelines for depression. The decision phase of the assessment encounter was not shown on the video. The participating IPs completed their medical disability reports, including the LFA, immediately after watching each client on the video. All reports and completed LFAs were collected directly afterwards. The researchers were blinded for the collection of data and an independent research assistant coded the data.

### Outcomes

The primary outcome of the RCT was guideline adherence, measured using performance indicators. A detailed description of the development and reliability of these performance indicators has been published elsewhere [11], as has the effect of the intervention on guideline adherence [4].

Secondary outcome in the RCT was LFA scores. These LFA scores were scored by the IP participating in the RCT for the work limitations of the clients presented in the four videos. The LFA consists of six sections containing a total of 106 items: I personal functioning (30 items), II social functioning (17 items), III adjusting to the physical environment (13 items), IV dynamic movements (31 items), V static posture (11 items), and VI working hours (4 items). A large-scale study (of 51,000 disability assessments) into the dimensions behind these items [12] discovered 16 dimensions, each forming an scale. The internal reliability of the scales (Cronbach’s alpha) was generally acceptable (alpha 0.60-0.75) to good (alpha >0.75) or even very good (alpha >0.85). Only one dimension – communication – had an unacceptable level of internal reliability (alpha 0.53). In a follow-up study using a second order factor analysis [13], 14 of these 16 scales (excluding communication and working hours) were further reduced to four scales:

1) Mental abilities: limitations in coping with various mental task demands

2) General physical abilities: limitations covering various aspects of the musculoskeletal system

3) Autonomy: limitations in being able to act autonomously in the working situation

4) Manual skills and grip strength limitations.

Since the internal reliability of this last scale was very low (alpha 0.46), items on this scale were included in the scale for general physical ability, a possibility demonstrated by another study of LFA data from 84,000 disability assessments [10]. The three scales in the mentioned study had an acceptable level of reliability (alphas were 0.69 for scale 1, 0.72 for scale 2, and 0.75 for scale 3 including manual skills and grip strength). Hence, in the current study we used these three scales, with an additional separate scale for working hours, that had a very good internal reliability (alpha 0.97) [12].

### Analyses

To address the first hypothesis, we used an unpaired t-test to analyse differences in the mean sum scores of the four scales between the intervention group and the control group for each case report (four case reports: the first two pre-intervention, the other two post-intervention). To examine whether correction was necessary for the influence of any unequal distribution of background variables between the intervention group and the control group, we performed regression analysis using the relevant background variable as covariate.

To address the second hypothesis regarding inter-rater reliability, we performed analyses using linear mixed models, which enable modelling of variances (and covariances) and provide the possibility of accounting for hierarchical data [14]. We used the variances to calculate the intraclass correlation coefficient (ICC, with values ranging between 0 and 1) [15]. A higher ICC is an indication of greater degree of inter-rater reliability. We also calculated whether the difference between the ICCs of the intervention group and the control group was significantly different from zero. For a more detailed description of the statistical analysis please we refer to the Additional file 1. All analyses were performed using SPSS 15.0 [14].

## Results

### Participants

Between January and March 2009 a total of 43 insurance physicians applied to take part in the course. At the time of the RCT all participating IPs were actively conducting disability assessments. Twenty-one IPs were allocated to the control group and 22 to the intervention group. One of the IPs who was allocated to the intervention group withdrew from the course and 2 IPs who were originally allocated to the control group were not available on the planned dates. All three were excluded from the RCT. At baseline, therefore, the control group (CG) consisted of 19 IPs and the intervention group (IG) of 21. For one CG participant and for one IG participant the LFAs of the two case reports after training were not available.

The separate baseline characteristics were equally distributed across both groups, apart from one variable (see Table 1). Although the mean number of clients with depression assessed by an IP per month was significantly higher in the CG, regression analysis demonstrated that this variable had no major effect on the magnitude of the sum scores of the four scales in the CG and IG for the four separate case reports. Correction for this variable in the analyses was therefore not necessary.

**Table 1:** Baseline characteristics of insurance physicians in control group (CG) and intervention group (IG)

	**CG (n = 19)**	**IG (n = 21)**	
**Baseline characteristics**	**Mean (sd) or percentage**		**p-value**
Age in years	50.5 (6.7)	51.1 (6.2)	0.923
Male	47%	52%	0.752
Weekly working hours	31.8 (9.9)	31.1 (9.2)	0.819
Years working as physician	21.7 (6.4)	23.5 (5.1)	0.319
Registered as insurance physician	84%	86%	0.894
Years working as insurance physician	15.4 (8.1)	15.6 (7.9)	0.922
Number of clients with depression assessed per month	9.3 (5.6)	5.3 (3.7)	**0.011**
Assessment time for depressed clients (minutes)	136.3 (62.3)	153.7 (48.4)	0.343
Assessments under the new disability act	68%	52%	0.301
Employee of the Institute	79%	81%	0.874

Significant difference between both groups (p < 0.05) in bold.

Institute: the Dutch Institute for Employee Benefits Schemes.

### Outcomes

Table 2 shows the mean scale scores (with standard deviation) for each LFA scale and the corresponding sum scores of the scales for the first two case reports before training, for both the control group (CG) and the intervention group (IG). For case report 1, most participants filled in items on the scales for working hours and mental abilities. About half the participants filled in items on the scale for physical abilities. Hardly any items on the scale for autonomy were filled in. The means of the sum score did not differ significantly over the four scales between CG and IG (p = 0.229). For case report 2, again most participants filled in items on the scale for mental abilities. This was also mainly the case for the scale for working hours in the IG, but not in the CG: in the CG about half the participants filled in work limitations on this scale. For the scales for autonomy and physical abilities, participants in the IG filled in items about twice as often as those in the CG. In the IG the mean sum score over the four scales was significantly higher than in the CG (p = 0.013).

**Table 2:** Mean scale scores (sd) of LFA scales for two case reports before training*

	**Case report 1: CG**		**Case report 1: IG**		**Case report 2: CG**		**Case report 2: IG**	
	**N (n)**	**Mean (sd)**	**N (n)**	**Mean (sd)**	**N (n)**	**Mean (sd)**	**N (n)**	**Mean (sd)**
**Working hours**	19 (17)	3.68 (2.08)	21 (18)	4.48 (2.27)	19 (10)	2.32 (2.69)	21 (18)	3.95 (2.42)
**Autonomy**	19 (2)	0.11 (0.32)	21 (1)	0.10 (0.44)	19 (5)	0.68 (1.45)	21 (11)	0.81 (1.03)
**Physical abilities**	19 (8)	1.53 (2.27)	21 (10)	1.95 (3.11)	19 (2)	0.32 (1.16)	21 (6)	1.48 (3.33)
**Mental abilities**	19 (19)	6.74 (3.18)	21 (20)	8.00 (3.96)	19 (19)	9.00 (3.80)	21 (21)	11.24 (4.07)
**Sum score**	19 (19)	12.05 (5.10)	21 (20)	14.52 (7.34)	19 (19)	12.32 (4.80)	21 (21)	17.48 (7.37)

* *LFA* List of Functional Abilities, *N* number of insurance physicians, *n* number of insurance physicians who filled in disabilities for (the scale of) the LFA, *sd* standard deviation. The difference of the mean sum scores over the four LFA scales between control group (CG) and intervention group (IG) is not significant for case report 1 (p = 0.229), but is significant for case report 2 (p = 0.013).

For the two case reports after intervention (case reports 3 and 4, see Table 3), the mean sum scores in the IG were significantly higher than in the CG (p = 0.023). For case report 3 few participants filled in items on the scales for autonomy and physical abilities. For case report 4 the tendencies and distribution of the CG and IG were only of interest for the scale for mental abilities. Here again the mean sum score in the IG was significantly higher than that in the CG (p = 0.04).

**Table 3:** Mean scale scores and sum scores of LFA scales for two case reports after training*

	**Case report 3: CG**		**Case report 3: IG**		**Case report 4: CG**		**Case report 4: IG**	
	**N (n)**	**Mean (sd)**	**N (n)**	**Mean (sd)**	**N (n)**	**Mean (sd)**	**N (n)**	**Mean (sd)**
**Working hours**	18 (6)	1.11 (2.27)	20 (16)	3.80 (2.33)	18 (0)	0.00 (0.00)	20 (5)	0.60 (1.14)
**Autonomy**	18 (3)	0.39 (0.98)	20 (3)	0.30 (0.80)	18 (0)	0.00 (0.00)	20 (0)	0.00 (0.00)
**Physical abilities**	18 (0)	0.00 (0.00)	20 (4)	1.25 (3.02)	18 (0)	0.00 (0.00)	20 (0)	0.00 (0.00)
**Mental abilities**	18 (16)	4.94 (3.81)	20 (19)	8.70 (3.80)	18 (16)	4.00 (2.38)	20 (20)	5.45 (2.42)
**Sum score**	18 (16)	6.44 (6.25)	20 (20)	14.05 (6.44)	18 (16)	4.00 (2.38)	20 (20)	6.05 (2.87)

* *LFA* List of Functional Abilities, *N* number of insurance physicians, *n* number of insurance physicians who filled in disabilities for (that scale of) the LFA, *sd* standard deviation. The difference of the mean sum scores over the four LFA scales between control group (CG) and intervention group (IG) is significant for both case report 3 (p = 0.001) and case report 4 (p = 0.023).

Table 4 shows the results of the mixed models analysis (parameters and standard errors), and of the ICC calculation for the presented case reports before training (case reports 1 and 2) and after training (case reports 3 and 4). For the case reports before training (case reports 1 and 2) the ICCs were similar (0.65 for the CG and 0.64 for the IG). For the case reports after training (case reports 3 and 4) the ICC in the IG was 0.69 and the ICC in the CG was 0.54. Upon testing, however, both differences in the ICCs between the IG and the CG were not significantly different from zero. The difference in ICC between the IG and CG (95% confidence interval) was: -0.01 (−0.56; 0.54) for case reports 1 en 2, and 0.14 (−0.35; 0.68) for case reports 3 and 4.

**Table 4:** Results of the mixed models analysis and ICC calculation, with scores of four LFA scales*

	**Case reports 1 and 2**		**Case reports 3 and 4**	
	**CG**	**IG**	**CG**	**IG**
Residual	5.28 (0.07)	6.37 (0.82)	2.52 (0.35)	4.06 (0.54)
Case report	0.00 (0.00)	0.00 (0.00)	0.00 (0.00)	0.00 (0.00)
Scale (case report)	10.29 (5.65)	14.64 (7.99)	3.89 (2.16)	9.98 (5.45)
Respondent	0.32 (0.34)	1.61 (0.84)	0.43 (0.35)	0.28 (0.36)
Case report * respondent	0.00 (0.00)	0.18 (0.60)	0.34 (0.34)	0.25 (0.43)
ICC	0.65	0.64	0.54	0.69
(95% confidence interval)	(0.33-0.84)	(0.32-0.83)	(0.21-0.76)	(0.37-0.86)

* Estimated for the case reports before training (case reports 1 and 2) and after training (case reports 3 and 4) in control group (CG) and intervention group (IG), with linear mixed models (parameters standard errors) and variance components for mixed models (ICCs and 95% confidence interval); the four disability scales are: working hours, autonomy, physical abilities, and mental abilities; *ICC* Intraclass Correlation Coefficient.

To determine whether the difference in ICCs between the CG and the IG as shown in Table 4 might have been influenced by the low number of observations for some of the abilities scales, the same analysis was conducted using either three scales – excluding the scale for physical abilities – or using two scales, i.e. using only the scales for working hours and mental abilities. Once more, the ICCs of the IG and the CG (see Table 5) were about the same for case reports 1 and 2 and higher for case reports 3 and 4 (0.21 higher when using 3 scales and 0.16 higher when using 2 scales). The differences in ICC between the IG and the CG were again in all cases not significantly different from zero (not shown here).

**Table 5:** Results of the ICC calculation, with scores of three and two LFA scales respectively*

	**Case reports 1 and 2**		**Case reports 3 and 4**	
	**CG**	**IG**	**CG**	**IG**
ICC (3 scales)	0.65	0.71	0.51	0.72
ICC (2 scales)	0.49	0.51	0.46	0.62

* Estimated for the case reports before training (case reports 1 and 2) and after training (case reports 3 and 4) in control group (CG) and intervention group (IG), with variance components for mixed models; the three disability scales are: working hours, autonomy, and mental abilities; the two disability scales are: working hours and mental abilities; *ICC* Intraclass Correlation Coefficient.

## Discussion

### Main findings

The results of this study show that before training the sum scores for the first case report did not differ significantly between the groups, while for the second case report the mean sum score was significantly higher in the IG than in the CG. For the two case reports after training, we saw a significantly higher score in the IG than in the CG.

The inter-rater reliability measured for the two case reports before training and using four scales was about the same in the CG and the IG. For the two other case reports after training, the ICC was 0.69 for the IG and 0.54 for the CG. This difference was not significant however.

### Interpretation and comparison with other studies

The training programme on applying the guidelines for depression resulted in more work limitations. For the same case report, IPs who received training filled in more work limitations in the LFA than the IPs who did not receive training. This difference is most noticeable in case report 3.

Post-intervention data showed that the group of IPs who were given training in applying the guidelines had a higher degree of consistency when filling in the LFA than the IPs in the control group. Apparently the implementation strategy contributed to more uniformity in work limitations assessments by IPs. This ties in well with earlier research into variation in work disability assessments [16,17]. In terms of financial and social consequences, such variation is unwanted for both the client and society and in our opinion might be reduced by the use of standardised methods of assessment, as occurs when guidelines are applied. The fact that applying guidelines results in a more uniform judgment ties in well with the idea that reducing medical ambiguity or uncertainty also reduces variation between doctors [18,19].

It is striking that the differences between the two groups with regard to the scale for working hours are considerable (except for case report 1), both before and after training. Working hours limitation is a strong determinant for the end result of the assessment: the degree of work disability assigned to the client. Another study into variations in disability assessments had also found little consistency between IPs regarding the work limitation scale for working hours [17]. The scale for working hours even has its own guidelines, separate from those specific to diagnosis [20].

Our results confirm the trends posed in the two hypotheses. We have shown that IPs trained in using the guidelines apply more work limitations than untrained IPs. In another study of ability assessments of clients with depression, the use of a work ability checklist actually led to findings of higher levels of work ability, without a reduction in the variation of assessment results [21]. One possible explanation for this is that the emphasis in the aforementioned study was on work ability rather than on work limitations as in the depression guidelines. Incidentally, the ICC in that study was of the similar magnitude to that found in the current study’s pre-intervention measurements, namely 0.64.

The training programme taught the IPs to conduct systematic and thoroughly justified disability assessments in accordance with the guidelines. Apparently this method of assessment leads to a higher number of work limitations than is usually the case. The reason for this might be that IPs who adhere more closely to guidelines interpret the information provided more strictly than usual. After all, the information concerning the client was provided by means of a case report on video, which was the same for all IPs. The IPs themselves were not able to ask the client any questions. Therefore, in the daily practice of IPs – where interviews form an influential part of a disability assessment – the difference between the groups may well be greater: the trained IP, actively applying the guidelines, will make further enquiries of the client regarding aspects such as sleep disorders. The existence of sleep disorders may then in turn influence how the IP fills in the LFA.

### Strengths and weaknesses

This study has several strengths. Firstly, the active form of the four ‘real life’ case reports on video, which simulate the daily practice of an IP, is more effective than written case reports [22]. Secondly, the fact that the two case reports presented before the training programme were different to the two after training prevents any confounding learning effect that occurs when a case report is presented for the second time. Thirdly, the suitability of the four scales drawn up on the basis of the LFA scientific research has already been established by statistical analysis in previous studies [10,12,13]: the difference in the means has been tested using the sum scores of the four scales, which are a valid measure of the number and severity of the limitations, since they are not influenced by the distribution over the four scales. Finally, to determine inter-rater reliability, an empirically tested method was used to calculate the ICCs (see Additional file 1): the differences between the ICCs of the CG and IG were tested for their significant difference from zero.

The study also has a number of weaknesses. To start with, it may be difficult for IPs to complete an LFA based purely on a video, a factor that was not looked at in this study. Another weakness is the question of what to do about items marked as ‘no limitations found’: should this be considered as missing data, or as an actual assessment of there being no limitations, or at least no severe limitations? We attempted to accommodate this weakness by also analysing inter-rater reliability while excluding the scales that had only a few observations. A further weakness is the fact that the pre-intervention data already showed a significant difference in the severity and number of limitations between the intervention group and the control group. Finally, since the case reports presented before and after the training programme were not necessarily comparable, the ICCs from before and after training were not comparable within each group (CG and IG). It was, therefore, not possible in the IG to test whether there was an increase in inter-rater reliability after the training programme.

### Practical relevance

The findings of this study provide a point of consideration for insurance medicine. IPs should be aware of the fact that collecting information about a client in a structural manner, as when following a guideline, can lead to the finding of more work limitations in that client. The IP should not lose sight of the importance of work participation and should focus on the work ability of the client. In addition, it would appear that IPs have difficulty reaching uniformity in applying the ‘reduced working hours’ standard [20]. We recommend a separate training programme for IPs to teach them to apply this standard, preferably according to the existing disease-specific guidelines.

Policy makers should be aware that although it is possible to improve the inter-rater reliability between IPs for disability assessments, there is still space for professional autonomy and variation in assessments, even after guidelines have been implemented. IPs cannot be completely constrained to a guideline and a guideline cannot be fully comprehensive to cover all possible situations. This study found a maximum ICC of 0.69, and not of 1.00. Since disability assessments are, and will remain, human activities, a certain degree of variation within professional guidelines is acceptable.

## Conclusion

There are indications that the implementation of a specially designed training programme on guidelines for depression may lead to greater inter-rater reliability in the assessments by insurance physicians of the work limitations of clients with depression. It is, however, important to note that insurance physicians who receive training may find more work limitations than those who do not. Whether this possible rise in work limitations found might also lead to a higher degree of work disability requires further investigation.

## Competing interests

The authors declare that they have no competing interests.

## Authors’ contributions

The authors declare that they participated in the study and made the following contributions to the study. AJMS, FZ, JRA and AJvdB contributed to the conception and design of this study. AJMS and FZ contributed to the analysis. AJMS and FZ wrote the manuscript. JRA and AJvdB revised and commented on the manuscript. AJMS and AJvdB will act as guarantors of this study. AJvdB had full access to all data in the study and had final responsibility for the decision to submit for publication. All authors read and approved the final manuscript.

## Supplementary Material

Additional file 1 Statistical method for calculating intraclass correlation coefficients. Click here for file
