# Supplementary material for: The influence of applying insurance medicine guidelines for depression on disability assessments
Source: BMC Res Notes. 2013 Jun 7;6:225. doi: 10.1186/1756-0500-6-225 (PMC3701610; doi:10.1186/1756-0500-6-225)
Supplement: Additional file 1 — Statistical method for calculating intraclass correlation coefficients. [file 1756-0500-6-225-S1.doc]

**Additional file 1: Statistical method for calculating intraclass correlation coefficients**

The intraclass correlation coefficients (ICCs) were calculated separately for the intervention group and the control group, both for the two case reports before the intervention and for the two case reports after the intervention. For this calculation we used a formula derived from the so-called generalisability theory [A1, A2], with the following variance components of a linear mixed model [A3, A4]: the two case reports, the four LFA scales nested within the case reports, the IPs, the interaction between the case reports and the IPs, and the residual variance. For the ICC calculation, the sum of the variance components for the case reports and the scales within the case reports formed the universe score, while the sum of the other variance components formed the absolute error variance. The ICC is defined as the ratio of the universe score to the sum of the universe score and the absolute error variance. The 95% confidence intervals were calculated from the variance components for a mixed model [A3] using Fisher’s Z transformation and the delta method [A5]. These methods were also used to calculate whether the difference between the ICCs of the intervention group and the control group was significantly different from zero (for a 95% confidence interval).

**References in Additional file 1**

A1. Shavelson RJ, Webb NM, Rowley GL: **Generalizability theory.** *American Psychologist* 1989, **44**:922–932.

A2. Shavelson RJ, Webb NM: **MMSS generalizability theory: a primer.** Newbury Park, CA: Sage 1991.

A3. **SPSS 15.0 Command Syntax Reference 2006.** Chicago III: SPSS Inc; 2006.

A4. Molenberghs G, Laenen A, Vangeneugden T: **Estimating reliability and generalizability from hierarchical biomedical data.** *J Biopharm Stat* 2007, **17**:595–627.

A5. Euser AM, Le Cessie S, Finken MJ, Wit JM, Dekker FW: **Reliability studies can be designed more efficiently by using variance components estimates from different sources.** *J Clinic Epidemiol* 2007, **60**:1010–1014.
